# Community-Based Active Tuberculosis Case Finding in Poor Urban Settlements of Phnom Penh, Cambodia: A Feasible and Effective Strategy

**DOI:** 10.1371/journal.pone.0092754

**Published:** 2014-03-27

**Authors:** Natalie Lorent, Kimcheng Choun, Sopheak Thai, Tharin Kim, Sopheaktra Huy, Reaksmey Pe, Johan van Griensven, Jozefien Buyze, Robert Colebunders, Leen Rigouts, Lutgarde Lynen

**Affiliations:** 1 Infectious Diseases Department, Sihanouk Hospital Center of HOPE, Phnom Penh, Cambodia; 2 Mycobacteriology Laboratory, Sihanouk Hospital Center of HOPE, Phnom Penh, Cambodia; 3 Mycobacteriology Unit, Institute of Tropical Medicine, Antwerp, Belgium; 4 Clinical Sciences Department, Institute of Tropical Medicine, Antwerp, Belgium; 5 Epidemiology and Social Medicine, University of Antwerp, Antwerp, Belgium; 6 Department of Biomedical Sciences, University of Antwerp, Antwerp, Belgium; National Institute of Infectious Diseases, Japan

## Abstract

**Background:**

In light of the limitations of the current case finding strategies and the global urgency to improve tuberculosis (TB) case-detection, a renewed interest in active case finding (ACF) has risen. The WHO calls for more evidence on innovative ways of TB screening, especially from low-income countries, to inform global guideline development. We aimed to assess the feasibility of community-based ACF for TB among the urban poor in Cambodia and determine its impact on case detection, treatment uptake and outcome.

**Methods:**

Between 9/2/2012-31/3/2013 the Sihanouk Hospital Center of HOPE conducted a door-to-door survey for TB in deprived communities of Phnom Penh. TB workers and community health volunteers performed symptom screening, collected sputum and facilitated specimen transport to the laboratories. Fluorescence microscopy was introduced at three referral hospitals. The GeneXpert MTB/RIF assay (Xpert) was performed at tertiary level for individuals at increased risk of HIV-associated, drug-resistant or smear-negative TB. Mobile phone/short message system (SMS) was used for same-day issuing of positive results. TB workers contacted diagnosed patients and referred them for care at their local health centre.

**Results:**

In 14 months, we screened 315.874 individuals; we identified 12.201 aged ≥15 years with symptoms suggestive of TB; 84% provided sputum. We diagnosed 783, including 737 bacteriologically confirmed, TB cases. Xpert testing yielded 41% and 48% additional diagnoses among presumptive HIV-associated and multidrug-resistant TB cases, respectively. The median time from sputum collection to notification (by SMS) of the first positive (microscopy or Xpert) result was 3 days (IQR 2–6). Over 94% commenced TB treatment and 81% successfully completed it.

**Conclusion:**

Our findings suggest that among the urban poor ACF for TB, using a sensitive symptom screen followed by smear-microscopy and targeted Xpert, contributed to improved case detection of drug-susceptible and drug-resistant TB, shortening the diagnostic delay, and successfully bringing patients into care.

## Introduction

Despite the progress made in the last decade, Cambodia remains one of the countries with the highest tuberculosis (TB) burden in the world [1]. TB case notifications - which continue to rely heavily on symptomatic individuals voluntarily seeking care at health facilities as advocated by the World Health Organisation – have stagnated. Several prevalence surveys, including Cambodia's latest, revealed up to 50% of tuberculosis remains undiagnosed despite widespread implementation of DOTS [2]–[6]. This “passive” facility-based case detection has proven inadequate to control TB [1], [5], [7].

TB tends to concentrate in poor and marginalised communities who face many barriers to access health services such as lack of awareness, competing priorities for time and money, disconnection with health services due to lack of regular service and experienced personnel [8]–[10]. Innovative strategies complementing facility-based case detection are needed. One such strategy, active case finding (ACF) – which involves systematically searching for TB in individuals who would not spontaneously present to a health service, and bringing them into care - has gained interest in high prevalence countries in the last decade [11]–[13]. ACF aims to reduce barriers for early TB case detection, including delay in presentation to a health facility, identification of a person as a presumptive TB case, and timely diagnosis and subsequent treatment. While showing significant promise as a tool to improve and accelerate TB diagnosis, subsequent treatment and cure must also be ensured to be effective and have an impact on TB transmission in the community [13]. Linking diagnosis to treatment has reportedly been challenging in community-based TB screening interventions with high initial loss to follow-up rates [11], [14], [15]. In places with an existing strong DOTS system, treatment outcomes were good [16]–[19]. So far, little is understood about how to best apply and integrate ACF in the existing health care systems in diverse epidemiologic, socio-economic, and cultural contexts [7], [20], [21].

Early detection of TB requires appropriate diagnostic tools. In many high burden countries smear microscopy remains the cornerstone of diagnosis. More sensitive and rapid diagnostics have become available but an affordable and accurate point-of-care test for TB is still lacking [22]. The revolutionary development of the GeneXpert MTB/RIF assay (further referred to as Xpert), a rapid and fully automated molecular test that simultaneously detects TB and rifampicin resistance, has definitely been a game-changer in this field. Endorsement of the test by the World Health Organisation has been quick [23] but financial and logistical constraints prohibit widespread use of the test in TB endemic low-resource countries. At the time, limited evidence existed on where to best position Xpert in the diagnostic algorithm [24]. Except one study from Lawn *et al.* on routine screening for HIV-associated TB before antiretroviral therapy [25], no data nor recommendations existed on the use of Xpert in ACF; few have been published since [26]–[28]. In a recent publication on systematic screening for active TB, the World Health Organisation addresses the issue and provides key recommendations on risk groups to be screened and algorithms (including Xpert) that can be used in ACF [29]. Although an important step in guiding screening for active TB and very helpful to potential implementers, the document also calls for more research on new screening approaches and tools, and more evidence on the impact of screening.

This study was undertaken to evaluate the feasibility of implementing community-based ACF using fluorescence microscopy and targeted Xpert in poor urban settlements of Phnom Penh, and determine its impact on TB case detection, treatment uptake and outcome.

## Methods

### Ethics statement

Ethical approval was granted by the National Ethics Committee for Health Research (Cambodia), and the Institutional Review Board at the Institute of Tropical Medicine and the Ethics Committee of the University Hospital, in Antwerp (Belgium). We also obtained permission from the national TB programme, the Municipality of Phnom Penh and the local health authorities. All participants provided written informed consent.

### Study site and setting

Cambodia, a low-income country in South-east Asia, has an estimated TB prevalence (all forms) of 817/100.000 population - one of the highest in the world - and an incidence of 411/100.000 population [1], [30]. Phnom Penh, the capital, had a population of 1.156.466 in 2010, with an estimated 30% living on less than US$ 2 a day [31].

Health services are organized into operational health districts. Each health district has a referral hospital and covers a number of health centres. Health centres provide basic care including TB services. Sputum smear microscopy and (if available) chest radiography are done at the referral hospital. Besides the four district hospitals, there are five tertiary hospitals in Phnom Penh providing care to patients from all over the country.

**Figure 1:**
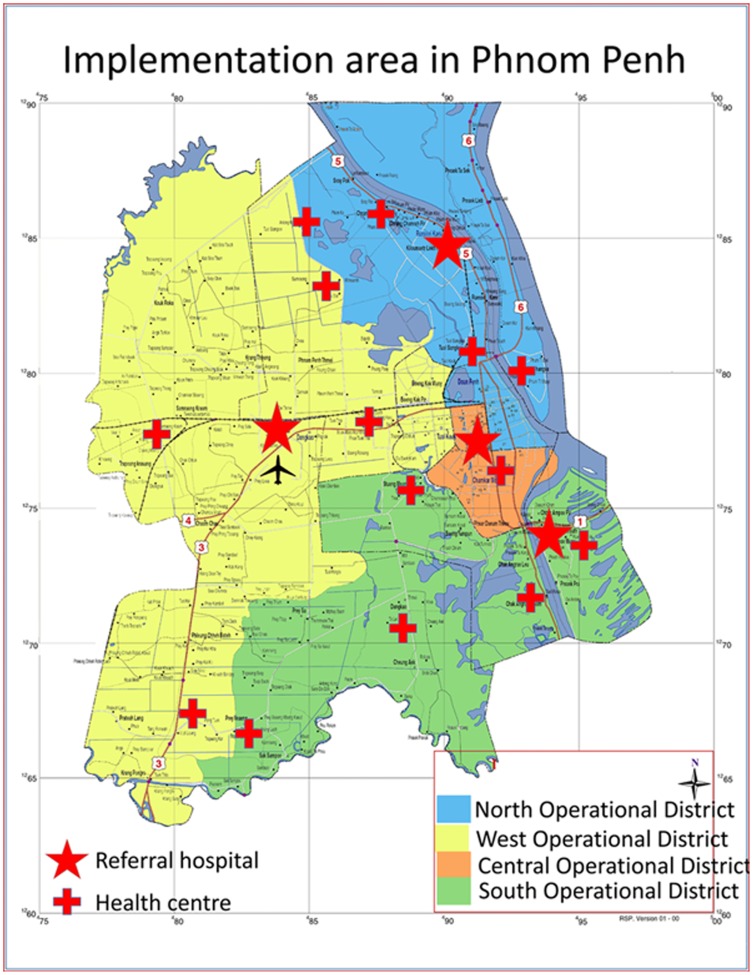
Map of the implementation area in Phnom Penh, indicating the health centers and referral hospitals located in the four operational districts.

Through a grant obtained from the TB REACH initiative (wave 2), the Sihanouk Hospital Center of HOPE conducted door-to-door screening for active TB in poor urban settlements of Phnom Penh from 9 February, 2012 to 31 March, 2013. We targeted communities with a presumed high prevalence of undiagnosed TB and/or restricted access to TB services. These included a heterogeneous group of slum dwellers, dump-site communities, migrants, factory workers, and displaced populations, the majority living in informal temporary or unstructured settlements. We selected the communities through purposeful sampling after consultation with health managers and municipal authorities. Community leaders, who were willing to participate, then identified the poorest or most hard-to-reach. The study area comprised 372 communities - scattered over the whole of Phnom Penh - with an estimated total population of 346.000. The study was embedded in the existing health services. It involved all levels of TB care of the four operational health districts of Phnom Penh providing services through 14 health centres, three referral operational district hospitals, and one tertiary level hospital (Figure 1).

### Intervention (Figure 2)

**Figure 2:**
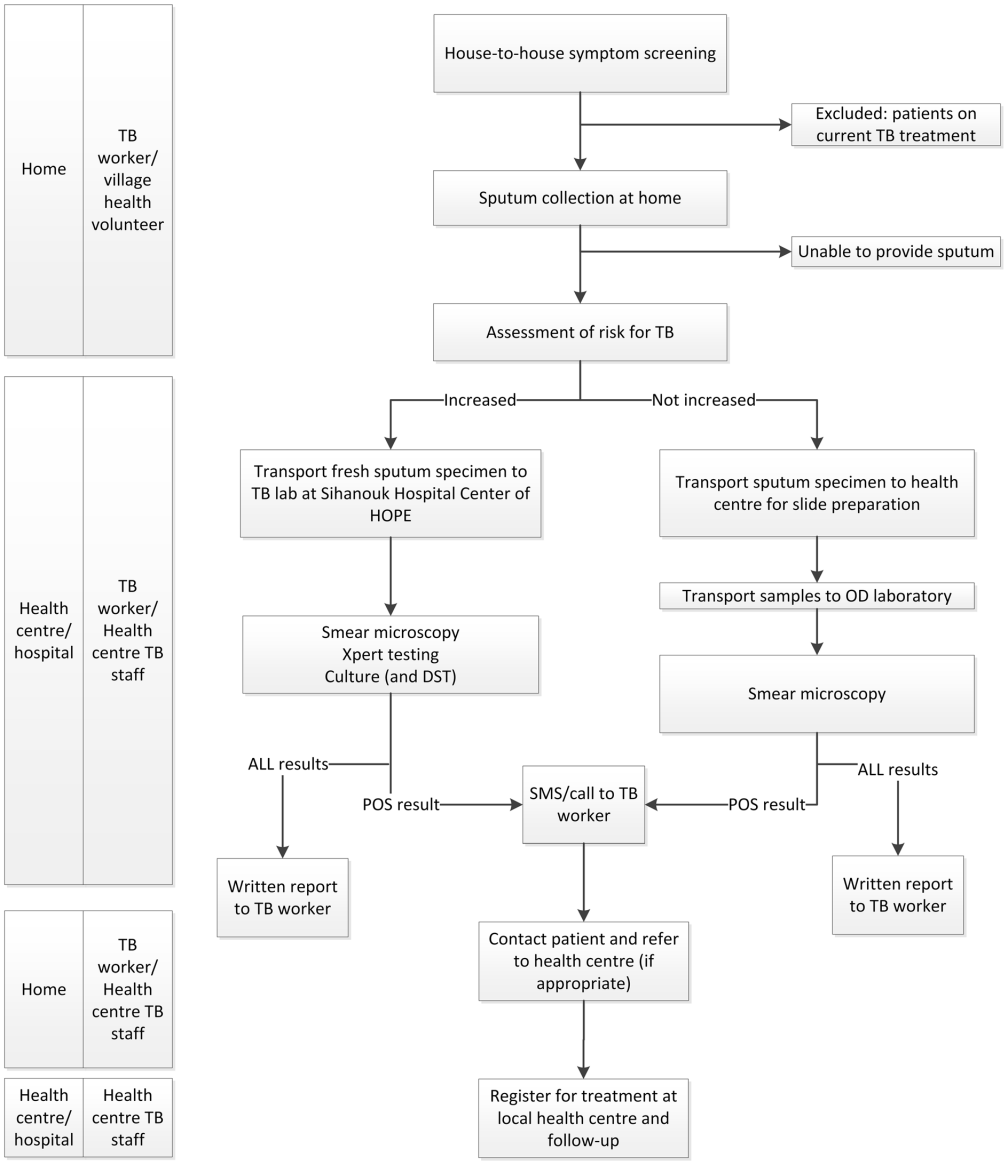
Schematic representation of the community-based active TB case finding approach. TB risk assessment: “high risk” (patients with previous TB history or contacts of known multidrug resistant tuberculosis cases; known or presumed HIV-infection; individuals with persistent symptoms suggestive of tuberculosis despite a negative smear) vs. no increased risk (none of the above; general (poor) population). SMS: short message system, TBW: TB worker.

Key components of the intervention were:

#### Screening

Trained TB workers, assisted by community health volunteers performed door-to-door visits to screen all available household members for symptoms suggestive of TB, defined as any cough, unintentional weight loss, fever, night sweats or haemoptysis. We relied on hetero-anamnesis for household members not at home at the time of the interview. If symptoms were reported, a new visit was arranged. If no symptoms were reported or the individual did not show up at the arranged visit no further attempts were made. All consenting adults aged 15 or more with a positive TB symptom screen (any of the above) were interviewed using a standardised questionnaire in the local language. Cases detected during the survey who had already started TB treatment were not considered as actively detected cases.

#### Sputum collection

The national TB programme recommends all symptomatic individuals to be evaluated with three sputum specimens. TB workers collected the specimens at home according to the following scheme: spot-spot-morning (SSM). Patients were instructed on how to produce a good quality sputum specimen following a standard operating procedure [32]. Ideally, the two spot specimens were collected the same day with approximately 1 hour interval, followed by an early morning specimen the next day. Specimens were transported daily to the health centre, where all cases were registered in the “sputum” registry and health centre staff prepared the slides. Subsequently, TB workers facilitated the transport of slides and specimens to the operational district referral laboratory as indicated. Fresh specimens for Xpert and culture (when indicated) were packed in secured, cool boxes and brought to the Sihanouk Hospital Centre of HOPE.

#### Clinical algorithm

As per national policy, microscopic examination of three smears was done for all patients. At the time of protocol development, other than the rapid implementation guide for Xpert [23], there were no (inter)national guidelines on how best to use Xpert for screening purposes. In our ACF programme Xpert indications were restricted to individuals with increased risk of TB and/or multidrug-resistant TB. More specifically, Xpert was done - in parallel with smear - for confirmed or presumptive (defined as chronic diarrhoea, cachexia and/or presence of papular pruritic eruptions) HIV-infected individuals, individuals with a TB history and symptomatic contacts of confirmed multidrug-resistant TB cases. In addition, targeted Xpert was done as an add-on test for presumptive smear-negative TB cases whose symptoms persisted after one week (regardless of antibiotics). For logistical and financial reasons we did not perform screening by chest radiography. If patients were unable to provide sputum they were referred to the health centres for further assessment as per national policy.

#### Laboratory procedure

Light emitting diode (LED)-based fluorescence microscopy (iLED Primostar, Zeiss, Germany) was introduced at three referral hospitals and one GeneXpert MTB/RIF platform (Cepheid SAS, Sunnyvale, USA) was acquired at the tertiary referral laboratory. Direct examination for acid-fast bacilli was done with LED fluorescence microscopes using auramine O-staining and potassium permanganate counterstaining. Except when fresh specimens were needed (for Xpert and/or culture), smear preparation was done at the health centres; followed by smear staining and reading at all three referral laboratories.

Xpert testing was done as per manufacturer's instructions. A single Xpert assay was done on a random spot or morning specimen. Inconclusive results were repeated once. For rifampicin-resistant cases on Xpert testing, a MTBDR*Plus* assay (Hain Lifesciences GmbH, Nehren, Germany) was requested to rapidly confirm isoniazid and rifampicin resistance at the laboratory of the Institut Pasteur de Cambodge.

A single culture was performed for all patients with presumptive HIV-associated or MDR-TB. Culture and drug-susceptibility testing (DST) were done on conventional solid medium (Löwenstein-Jensen, LJ) following standard operating procedures of the laboratory. Cultures were considered negative if no growth was obtained after 8 weeks. Specimens yielding growth (any number of colonies) were subjected to MPT64-based species confirmation as per manufacturer instructions (SD Bioline TB Ag MPT64 Rapid-Alere, Standard Diagnostics, Inc., Korea). If growth yielded *Mycobacterium tuberculosis*, conventional proportion method DST (for first- and second-line drugs using standard critical concentrations: isoniazid 0,2 μg/ml and 1 μg/ml; rifampicin 40 μg/ml; ethambutol 2 μg/ml; streptomycin 4 μg/ml, ofloxacin 2 μg/ml and kanamycin 30 μg/ml) was performed. A para-nitro-benzoate test was included in the DST procedure.

All laboratories participated in external quality assurance for smear microscopy through the national TB programme. The Sihanouk Hospital Center of HOPE laboratory also participated in annual DST rounds by the Institute of Tropical Medicine (Antwerp) and passed a validation test for Xpert by the manufacturer.

#### Patient management and follow-up

As soon as available, laboratory technicians issued positive results from smear microscopy or Xpert by short message system (SMS) to the respective TB workers, who (ideally within 24 h) contacted the patient either directly or via the community health volunteer. Smear- or Xpert-positive TB patients were referred to the health centre for initiation of TB treatment at their earliest convenience. Written results were distributed to the TB worker at least once a week, including negative results. When smear microscopy was negative but TB symptoms persisted, individuals were advised to contact the TB worker or community health volunteer. They would then be asked to submit two more sputum specimens (for repeat smear and Xpert), and referred for further diagnostic work-up including chest radiography as per national policy.

#### TB treatment

First-line anti-TB drugs were started for all bacteriologically-positive cases as well as for individuals with a high clinical suspicion of smear-negative or extra-pulmonary TB according to the DOTS-strategy [33]. When rifampicin resistance was detected by Xpert, the patient was referred for standardised second-line treatment at one of the MDR-TB treatment sites; treatment was later adjusted to DST results if indicated. All patients starting TB treatment were offered free HIV counselling and testing.

TB workers and community health volunteers actively tracked patients who dropped out after diagnosis, or interrupted treatment and encouraged them to initiate or complete TB treatment. This could require several (up to three) visits. To ensure active engagement in care, the most convenient treatment modality (community- or ambulatory-DOTS), was discussed.

Treatment outcomes were abstracted from the health centre's treatment registers in the first place. If not found, TB workers enquired the patient or his/her relatives by phone or on house visit.

### Preparation and implementation process

We newly recruited all TB workers, some of (para-)medical background, and trained them on TB diagnosis and treatment as well as data collection. They received a mobile phone, an ice box and a back pack containing data collection tools and sputum cups. Community health volunteers - lay members, some of whom had previously participated in health projects - were assigned by the village leader. They accompanied and assisted TB workers on their rounds. Health centre TB staff were offered a refresher course on smear preparation. LED fluorescence microscopy was introduced at three referral laboratories. Laboratory personnel attended a one-week training by the national tuberculosis programme. To ensure quality microscopy services the Sihanouk Hospital Center of HOPE provided continued training and on-site support for laboratory technicians in the district hospitals, and monthly quality control checks on a random selection of ten smears. A core team from Sihanouk Hospital Center of HOPE met weekly to address issues regarding coordination, clinical or laboratory work, research, and data management and evaluate the progress. Separately, all field TB workers were offered the opportunity to provide feedback on their activities during weekly meetings with the coordinating team. The coordinating team and the operational health district's TB staff also met quarterly.

All TB workers received an appropriate salary and travel allowance. All community volunteers and health district personnel received a small incentive (US$ 15–30/month) to compensate for the extra work delivered to the project.

### Outcome measurements

Smear-positivity was defined as at least one smear containing at least one acid-fast bacillus [33]. All patients with a positive smear, or *M. tuberculosis* detected by Xpert or isolated by culture were considered bacteriologically-proven TB [34]. Clinical TB was defined as active TB diagnosed by a clinician or other medical practitioner who decided to give a full course of TB treatment based on clinical, radiology and/or histology findings but without bacteriological confirmation [34]. TB treatment was initiated based on the first available positive result or on clinical indication. Time to treatment initiation for bacteriologically-confirmed TB was defined as the interval between the first positive result and the start of treatment. Treatment outcomes - cured, treatment completed, treatment failed, died, lost to follow-up, not evaluated - were reported in line with the latest World Health Organisation's definitions [34]. Delay in linkage to care was defined as a failure of patients, identified through the survey, to start TB treatment within 1 week after diagnosis. Patients who failed to commence TB treatment were considered initial “lost to follow-up” (previously “initial defaulters”) [34], [35].

### Statistical analysis

Data were entered in an Access database. Verification of original data (including patient questionnaires, laboratory registers, health centre registers) was done monthly for a random sample of ten TB patient files. Error queries were run weekly to check for missing data and resolve inconsistencies.

Patients' demographic and clinical characteristics were described in terms of percentages, medians and interquartile ranges (IQR). We calculated frequencies, proportions and 95% confidence intervals (95% CI). A two-sided p-value<0.05 was considered significant. All analyses were performed using STATA software version 10.0 (College Station, Texas, USA).

## Results

### Identification of symptomatic individuals and diagnosis

**Figure 3:**
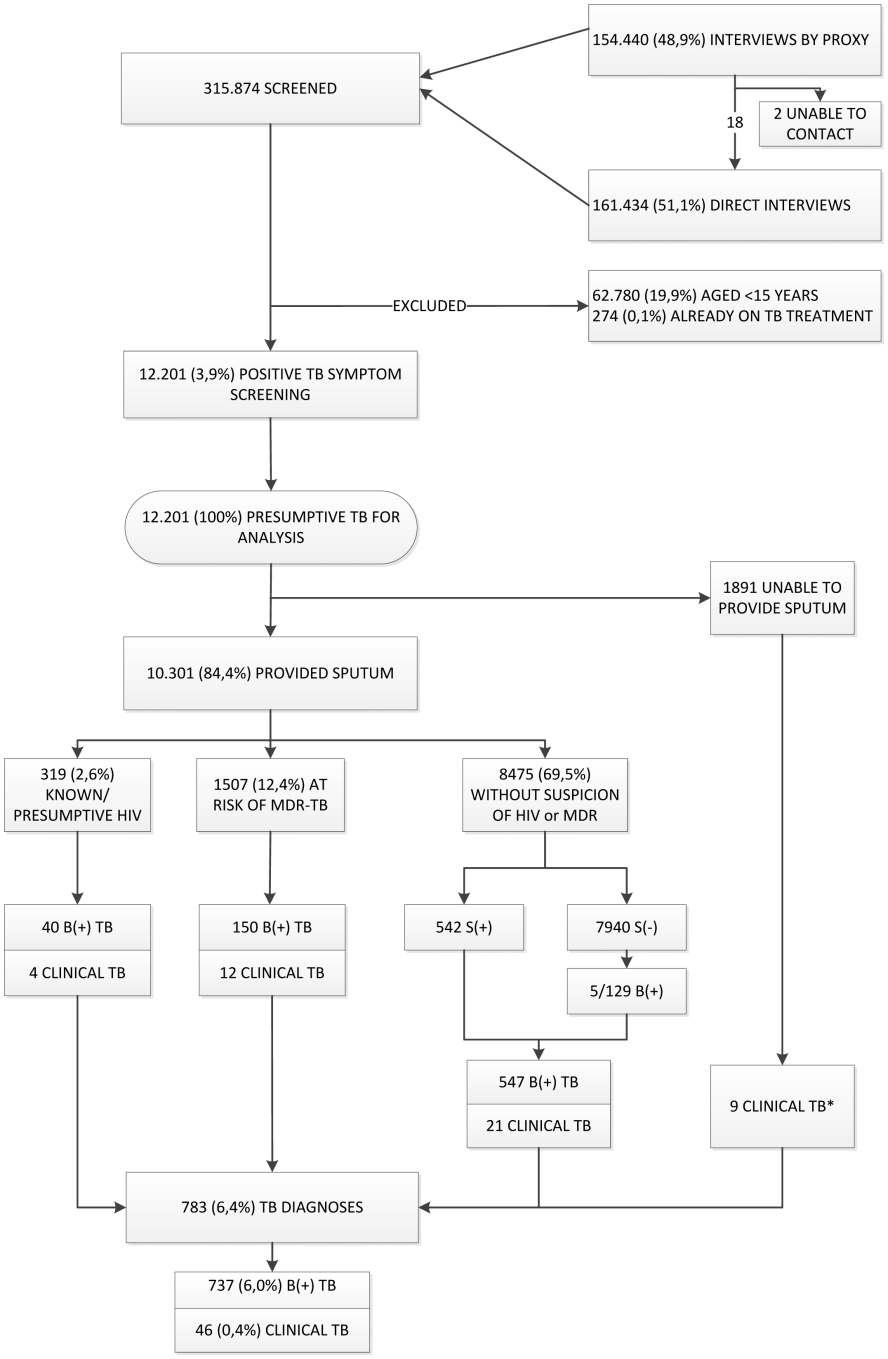
Study flow and tuberculosis yield for the different subgroups. Individuals not screened directly were screened verbally through a proxy. Presumptive tuberculosis indicates a positive symptom screen (any cough, fever, night sweats, weight loss or hemoptysis). *Of the patients who did not provide sputum, 2/114 patients at risk of MDR-TB were diagnosed with clinical TB (one with extra-pulmonary TB and one non-confirmed pulmonary TB), and 7/1.737 patients without risk for HIV-associated or MDR-TB were diagnosed with extra-pulmonary TB. HIV: human immunodeficiency virus; MDR-TB: multidrug-resistant tuberculosis; S(+): smear-positive; B(+): bacteriologically-positive; TB: tuberculosis.

Over 14 months, a team of 37 TB workers assisted by 372 community health volunteers screened a population of 315.874 (out of an estimated 346.000) for TB. Figure 3 shows the participant flow. We enumerated all household members and interviewed 161.434 (51,1%) directly (i.e. the participant was present at the time of the interview). Of the 154.440 interviewed through a proxy, 20 were reportedly symptomatic. We investigated 18 of them; two never showed up. For analysis we excluded 62.780 children (19,9%; 13 diagnosed with TB) and 274 (0,1%) individuals who were on TB treatment at the time of the screening (217 of the directly and 57 of the indirectly interviewed). We identified 12.201 individuals (3,9% of the screened population) with TB symptoms: 46,0% were male, the median age was 45 (IQR: 31–58) years, 11,2% previously had TB and 0,9% were known HIV-infected. The largest group comprised individuals who had no risk factors for HIV or MDR-TB (Table 1).

**Table 1:** Clinical characteristics of all individuals with presumptive TB, i.e. a positive tuberculosis symptom screening.

Variables	Total (n = 12.201)	Presumptive or confirmed HIV (n = 359)	Presumptive MDR TB (n = 1.623)	No HIV or MDRTB suspicion (n = 10.219)
Age (y), median (IQR)	45 (31–58)	45 (31–57)	54 (42–66)	44 (30–57)
Male gender, n(%)	5.611 (46,0)	176 (49,0)	776 (47,8)	4.659 (45,6)
HIV confirmed*, n(%)	11 (0,09)	11 (3,1)	-	-
Previous TB history, n (%)	1.368 (11,2)	3 (0,8)	1.365 (84,1)	0 (0)
Sputum provided, n (%)	10.310 (84,5)	319 (88,9)	1.509 (93,0)	8.482 (83,0)

HIV: human immunodeficiency virus, MDR-TB: multidrug-resistant tuberculosis.

*HIV confirmed refers to those with a known HIV-infection prior to TB diagnosis. An additional 11 were diagnosed HIV-positive after TB diagnosis: 2 individuals from the presumptive HIV-group, 3 from the MDR-TB risk group and 6 from the no-risk group.

Table 2 shows the diagnostic tests that were done on the 10.301 (84,4%) presumptive TB cases who provided sputum; 93,8% (n = 9.658) of whom gave at least two sputum specimens. A total of 1.828 individuals were eligible for Xpert as initial diagnostic test - in parallel with smear microscopy - because of confirmed/presumed HIV infection or risk of MDR-TB; 1.742 (95%) effectively received the test. A total of 129 of the 7.940 (1,6%) presumptive TB cases (at no increased risk) with initial negative smears returned for further diagnostic work-up and were eligible for an add-on Xpert testing; 97% (125/129) received the test. TB culture was done for 1.538 individuals.

**Table 2:** Tuberculosis diagnostics done for individuals with presumptive TB who were able to provide sputum.

Variables	Total (n = 10.310)	Presumptive or confirmed HIV (n = 319)	Presumptive MDR-TB (n = 1.507)	No HIV or MDR-TB suspicion (n = 8.475)
Smears done, n (%)	10.301	319	1.507	8.475
Smear result				
overall positive, n (%)	663 (6,4)	27 (8,5)	94 (6,2)	542 (6,4)
scanty, n (%)	340 (51,4)	14 (51,8)	39 (41,4)	287 (53,0)
1+, n (%)	122 (18,4)	2 (7,4)	16 (17,0)	104 (19,2)
2+, n (%)	79 (12,0)	4 (14,8)	11 (11,7)	64 (11,8)
3+, n (%)	122 (18,4)	7 (25,9)	28 (29,8)	87 (16,0)
negative	9.638 (93,6)	292 (91,5)	1.413 (93,8)	7.933 (93,6)
Xperts done, n (%)	1.894*	292	1.450	125
Xpert result, n (%)				
MTB+/RIF-, n (%)	135 (7,1)	28 (9,6)	94 (6,5)	5 (4,0)
MTB+/RIF+, n (%)	8 (0,4)	2	6	0
MTB+/RIF indeterminate, n (%)	6 (0,3)	1	5	0
MTB-/RIF-, n (%)	1.731 (91,4)	260 (89,0)	1.334 (92,0)	118 (94,4)
Inconclusive, n (%)	14 (0,7)	1 (0,3)	11 (0,8)	2 (1,6)
Cultures done, n (%)	1.538	227	1.308	3
Culture result, n (%)				
* M. tuberculosis*, n (%)	79 (5,1)	18 (7,9)	61 (4,7)	-
NTM, n (%)	12 (0,8)	-	12 (0,9)	-
sterile, n (%)	1.392 (90,5)	201 (88,5)	1.188 (90,8)	3*
contaminated, n (%)	55 (3,6)	8 (3,5)	47 (3,6)	-

*Due to clerical errors, 27 Xperts were done on presumptive TB cases non-eligible for Xpert; eight of those were positive; and three cultures were done, all sterile.

HIV: human immunodeficiency virus, MDR-TB: multidrug-resistant tuberculosis, MTB+: *M. tuberculosis* detected, MTB-: *M. tuberculosis* not detected; RIF-: rifampicin resistance not detected; RIF+: rifampicin resistance detected; NTM: non-tuberculous mycobacteria

### Yield of TB screening

Among individuals providing a sputum specimen (n = 10.301) 7,2% (95% CI: 6,7–7,7) had bacteriologically-positive TB. However, this ranged from 12,5% (40/319, 95% CI: 9,1–16,7) among the presumptive or confirmed HIV-infected patients, 10,0% (150/1.507, 95% CI: 8,5–11,6) among individuals with risk factors for MDR-TB and 6,5% (547/8.475, 95% CI: 5,9–7,0) in the group without any of these risk factors. The median age of patients diagnosed with bacteriologically-positive TB was 47 years (IQR 32–60); 55,4% were men.

Besides the 737 bacteriologically-confirmed TB cases, we diagnosed 46 “clinical” TB cases without confirmation (32 pulmonary and 14 extra-pulmonary TB) totalling up to 783 (6,4% of presumptive TB cases directly screened) adult TB diagnoses (Figure 3).

Considering all 315.874 individuals screened, overall TB prevalence was 339/100.000 (95% CI: 319–360) including 274 patients already on TB treatment and 13 children diagnosed with TB.

### Additional yield in terms of TB diagnosis and rifampicin resistance detection by Xpert compared to fluorescence microscopy

Of the 737 bacteriologically-confirmed TB cases, the majority (n = 663, 90%) could have been diagnosed by a positive smear only. Of the 10.301 presumptive TB patients whose sputum was analysed 663 (6,4%) had a positive smear; 48,7% of which were graded 1+ or more.

Table 3 shows the additional yield of and number needed to test for Xpert. In the HIV-group, Xpert confirmed 11 additional (initially smear-negative) TB diagnoses (41% increase) and detected 2 rifampicin-resistant smear-positive TB cases. Forty-five additional (initially smear-negative) cases were detected among patients at risk of MDR-TB, reflecting a 48% increase in TB case detection, and 6 rifampicin-resistant smear-positive cases in this subpopulation. For individuals at no increased risk and negative smears, Xpert increased case detection by 1% accounting for the fact that only 125 (1,6%) of the 7.933 returned for the add-on test. However, for smear-negative TB patients with persistent symptoms the number of Xperts needed to confirm one additional TB case was 26.

**Table 3:** Additional yield with regards to TB diagnosis and rifampicin resistance detection of Xpert compared to fluorescence smear microscopy.

Risk group	Xpert tests done	Additional TB diagnostic yield			Additional diagnosis of Rif-R	
		N	% increase	NNT	N	NNT
**Presumptive or confirmed HIV (smear/Xpert in parallel) n = 319**						
smear(+)	27	-	-	-	2	14
smear(−)	292	11	41% (38/27)	27	0	-
**Presumptive MDR-TB (smear/Xpert in parallel) n = 1.507**						
smear(+)	94	-	-	-	6	16
smear(−)	1.414	45	48% (139/94)	32	0	-
**Smear(-) with persisting symptoms (Xpert as add-on) n = 125/7.933 smear(-)**						
smear(+)	542	-	-	-	-	-
smear(−)	7.933	-	-	-	-	-
2^nd^ round smear(−)	125	5	1% (547/542)	26	0	-

Smear(+): smear-positive; smear(−): smear-negative; HIV: human immuno-deficiency virus; NNT =  number needed to test; Rif-R: rifampicin resistance; MDR-TB:multidrug-resistant tuberculosis.

Overall 61 additional TB cases were identified by Xpert and 13 by culture.

### Treatment uptake and outcome

The median time from sputum collection to the first positive result either by smear microscopy or Xpert was 4 days (IQR 2–7). Due to clerical errors only a limited number of SMS data (237/737, 32%) from a single laboratory were available. Positive results were sent by SMS at a median of 3 (IQR 2–6 for smear microscopy, IQR 2–7 for Xpert) days after sputum collection, whereas written results (including positive and negative results) were available at a median of 5 days (IQR 2–8).

Table 4 shows the treatment uptake and outcome. Of the 783 TB cases diagnosed, 741 (94,6%) begun TB treatment. The median time from sputum collection to TB treatment initiation was 8 (IQR 5–14) days. Treatment was initiated at a median of 3 days (IQR 1–6) from the first bacteriological confirmation of TB.

**Table 4:** Treatment uptake and outcome of TB patients.

Variables	Total (n = 783)	Presumptive or confirmed HIV (n = 44)	Presumptive MDR TB (n = 164)	No HIV or MDR-TB suspicion (n = 575)
**Treatment started, n (%)**				
confirmed	741 (94,6)	41 (93,1)	149 (90,8)	551 (95,8)
unknown (referred out before treatment)	21 (2,6)	2 (4,5)	8 (4,8)	11 (1,9)
never^a^	21 (2,6)	1 (2,3)	7 (4,3)	13 (2,2)
Median (IQR) time to treatment from screening	8 (5–14)			
Median (IQR) time to treatment after positive result	3 (1–6)			
**Time to treatment after diagnosis** b **(n = 739)**				
no delay (within 1 week) (n,%)	588 (79,5)	33 (84,6)	112 (75,2)	441 (80,0)
delay >1 week - <2 weeks	91 (12,3)	4 (10,2)	20 (13,4)	67 (12,2)
delay >2 weeks - <4 weeks	40 (5,4)	1 (2,6)	11 (7,4)	28 (5,1)
delay >4 weeks	20 (2,7)	1 (2,6)	6 (4,0)	13 (2,4)
**Treatment outcome of first-line TB regimen (n = 776)**				
treatment success	632 (81,4)	36 (85,7)	114 (71,7)	482 (83,8)
cured	536 (69,1)	28 (66,7)	75 (47,2)	433 (75,3)
completed	96 (12,4)	8 (19,0)	39 (24,5)	49 (8,5)
treatment failed	4 (0,5)	0	1 (0,6)	3 (0,5)
died	18 (2,3)	1 (2,4)	8 (5,0)	9 (1,6)
lost to follow-up^c^	59 (7,6)	3 (7,1)	18 (11,3)	38 (6,6)
not evaluated^d^	63 (8,1)	2 (4,8)	18 (11,3)	43 (7,5)
**Treatment outcome of second-line TB regimen (n = 7)**				
died	1	0	1	0
pending (treatment on-going)	6	2	4	0

a including two patients who died before treatment.

b diagnosis based on first positive sample for smear or bacteriologically positive TB cases or on clinical/radiological assessment for clinical TB diagnosis.

c lost to follow-up includes all TB patients who did not start treatment (n = 21) or whose treatment was interrupted for 2 consecutive months or more (n = 38).

d no treatment outcome assigned, including unknown or “transfer out” cases.

Eight MTB+/RIF+ cases detected (incl one failure of MDR-risk group); one patient refused second line drugs so her outcome is reported under first-line regimen as per WHO recommendations.

Twenty per cent experienced a treatment delay of more than one week. Another 21 (2,6%) TB patients never started treatment despite several counselling visits. This included two patients who died one month after diagnosis but who had not yet started treatment.

The overall treatment success rate of a first-line TB regimen was 81,4% (n = 632/776). Four individuals failed treatment, 18 (2,3%) died, 59 (7,6%) were lost to follow-up (including 38 who interrupted treatment and 21 who never started), and 63 (8,1%) were not evaluated because they were transferred out and/or treatment outcome was not reported. Of the eight patients with rifampicin resistance detected on Xpert, all but one were successfully started on a standardised second-line regimen. One subsequently died on treatment; other outcomes are pending.

### Yield in terms of case notifications

Case notifications in Phnom Penh increased from 4.073 to 4.561 – a 12% increase - for all forms of TB in the five quarters preceding ACF implementation (quarter four of 2010 plus 2011) compared with the intervention period (2012 and first quarter of 2013). Case notifications of bacteriologically-confirmed TB increased from 1.610 to 2.075 – a 29% increase - over the same period of time [36]–[38]. If we correct for the fact that these data were collected over five quarters - while we collected data in five quarters minus 39 days – our ACF-project contributed 19% of the case notifications of all forms and 39% of the bacteriologically-positive TB.

## Discussion

From February 2012 to March 2013 we performed door-to-door active TB screening in poor urban settlements of Phnom Penh reaching a population of 315.874. We diagnosed 783 TB cases – 737 of which were bacteriologically-confirmed - within a median of three days for smear- or Xpert-positive disease. Overall 95% begun TB treatment and 81% obtained a successful treatment outcome; less than 3% failed to start.

The effectiveness of community-wide active TB screening is widely debated for various reasons: the high number of individuals needed to screen, the risk of false-positive TB diagnoses and the unacceptable number of patients failing to start treatment [39], [40]. We indeed confronted most of these concerns, though with some consideration.

The latest national prevalence survey in Cambodia revealed a substantial burden of undetected TB, with TB prevalence rates more than twice as high as TB case notification rates particularly among persons aged more than 45. It also showed that the symptom screening used - cough >2–3 weeks or haemoptysis - had poor sensitivity: more than 70% of the smear-negative culture-positive TB cases were “asymptomatic” [30]. We therefore opted for large-scale simple and sensitive symptom screening. This resulted in high numbers needed to screen (NNS = 403), in line with estimates for community-wide screening from a systematic review by Shapiro *et al.* [29]. Although labour-intensive, our approach was feasible because we could rely on a well-functioning network of community health workers. Previously, Datiko *et al.* have shown the important role of community health workers by improving access to TB diagnosis and care in enhanced case finding in rural Ethiopia [41]. Given the achievements in several health domains, community health worker involvement is proliferating, also in Cambodia. For their successful contribution to be sustained, integration in the health system and formal recognition and remuneration must be pursued [42].

Xpert clearly outperformed smear microscopy in the few studies reporting on its use in ACF [25]–[28]. Xpert detected 73% of the active TB cases whereas microscopy detected 28% when used in systematic screening for TB among HIV-infected patients before the start of ART [25]. Among HIV-infected inmates in a Malaysian prison Xpert accurately identified 8/15 culture-confirmed TB; smear microscopy was positive in one [26]. In a large TB prevalence survey in a South-African gold mine, Dorman *et al*. also reported substantially higher performance characteristics for Xpert compared with microscopy (63% vs. 18% sensitivity) [27]. A small pilot study of ACF among smear-positive TB household contacts in Tanzania revealed the sensitivity of Xpert to be 40% higher than smear microscopy [28]. We performed Xpert for individuals at risk of HIV-associated or MDR-TB resulting in a 41 and 48% increase in TB diagnosis, respectively. Although encouraging, the utility of Xpert in active TB case finding in terms of performance, cost and impact requires further evaluation especially for clinical populations other than HIV-associated TB and/or risk of MDR-TB.

To achieve an impact on patients' outcomes and TB transmission, active screening for TB ideally engenders improved case detection, shortened diagnostic delays and earlier treatment initiation [40]. First, referring to previous years, case notifications in the whole of Phnom Penh increased during the implementation period. While ACF likely contributed, it is hard to assess its additionality given the lack of baseline data relevant to the target population. Also, a proportion of actively detected cases may have turned up for passive case finding possibly after a longer delay [43]. In this respect it is worth referring to smear-grades of our actively detected cases. On the one hand, a substantially higher proportion of positive smears were scanty in comparison with passive case- case finding (51 vs. 35%; source: laboratory reports, Sihanouk Hospital Center of HOPE) supporting previous reports that provider-initiated TB screening allows earlier identification of TB suspects [19], [44]. On the other hand, 30% of the cases detected had highly positive smears, as previously observed in Kenya and South Africa [6], [44]. This finding reflects the apparently low rate of detection, yet high rate of transmission and supports the complementary role of alternative case finding strategies, such as ACF, in Cambodia [45].

Second, we were able to issue results for bacteriologically-confirmed TB within a median of three days from sputum collection, which is a substantially shorter turn-around-time than the generally reported 7–10 days delay (community experience). The use of mobile phone/SMS contributed to expediting the time to diagnosis, as did other interventions such as facilitation of specimen transport, and not at least the active network of community workers, health centre staff and laboratory technicians. While we should aim for same-day issuing of results [46], the delay reflects the current reality on the ground and accounts for the late arrival of the specimens in the laboratory, at times (too) high work load in the laboratory, and reporting delays due to not-automated sending of SMS. This observation underlines the urgent need for a real point-of-care test for TB. Meanwhile, if infrastructure allows, further decentralisation of TB diagnosis could contribute to reducing diagnostic delays.

Third, the impact of ACF is thwarted when improving case detection without ensuring treatment initiation and completion. Unlike reports from South-Africa and India [11], [15], treatment uptake in our study was high. However, one in five patients delayed treatment for various patient- and health system-related reasons such as inability to find the patient, initial refusal of diagnosis, travel distance, need for hospitalisation, inconvenient opening hours of health facilities, or perceived cost of treatment. They might not have started treatment or only started at a more advanced stage of the disease were it not for the patient-centred services of community-health workers attending to their needs including intensive counselling, close patient follow-up, adherence support and removing financial and logistical barriers to treatment [47].

Taking into account that our population was highly mobile and hard-to-reach, the 2,6% initial lost to follow-up compares favourably with the 32% initial lost to follow-up in India [15]. It is similar to the 2,5% among household contacts actively screened in Cambodia [19].

Successful treatment outcomes of 81% are consistent with findings from ACF-projects in Cape Town [11] and Brazil [16]. They are substantially lower than the 96% reported in ACF among household contacts in Cambodia [19] or the more than 90% success rate of national TB treatment outcomes of passive case finding [19], [36] but contrary to the above, our outcomes include the loss to follow-up prior to treatment (as per latest outcome definitions of the World Health Organisation). Obtaining treatment outcomes of referred patients was problematic and will hopefully improve in the near future as the country will introduce electronic registration of all TB patients. There is also a need to explore more efficient strategies of engaging actively detected TB cases into care.

Although not without challenges, door-to-door screening in our communities was operationally feasible and effective. Different interventions for community TB screening have been described such as ACF through home visits [16], [17], [48] or mobile clinics [13], or enhanced case finding through home visits followed by referral to a health facility [18]. In the DETECTB-trial in Zimbabwe [13] the mobile van strategy outperformed door-to-door enquiry, whereas in the ZAMSTAR trial the household intervention proved more effective [12]. There might not be a single best strategy to fit all socio-cultural contexts for TB screening. Though assumed high, queries into the acceptability of TB screening are limited [29]. The acceptability of home visits in our setting is being evaluated in more depth through qualitative research and will inform implementation of future rounds.

### Limitations

Although our study has several strengths such as the large-scale implementation of community-based ACF embedded in a well-functioning national TB programme, low initial lost to follow-up rate and high treatment success, it also has limitations. Almost 50% of the household members in the communities were not available for interview, mostly because of work, and symptom screening was done through a proxy. This may have led to an underestimation of presumptive or real TB cases since no further investigations were done if an individual reportedly had no symptoms.

The current strategy resulted in an under-diagnosis of smear-negative TB: 6%, in contrast with 40% of smear-negative or extra-pulmonary TB cases notified annually to the national TB programme. The improved sensitivity of fluorescence microscopy and Xpert only could not explain this finding. Targets and incentives drove the case detection of bacteriologically-confirmed TB, likely overlooking presumptive TB cases who were unable to provide sputum or smear-negative but did not return for further work-up. A more comprehensive diagnostic algorithm including screening with CXR and ensuring diagnostic work-up of non-confirmed symptomatic individuals is recommended.

In 9,5% of the smear-positive patients with prior TB non-tuberculous mycobacteria grew on culture (and Xpert remained negative), suggesting that the concern of false-positive TB diagnosis (if only smear microscopy is used) is justified in certain subgroups. Further research on the confounding role of underlying lung disease on TB diagnosis in ACF is warranted.

Although our study is one of the few large-scale ACF studies using targeted Xpert (and culture), the majority of TB cases was diagnosed by smear microscopy. Xpert and culture were performed for high-risk patients only on randomly selected specimens with possible different bacterial load. Hence, a discussion on sensitivity and specificity of Xpert and culture in ACF is beyond the scope of this study.

No formal cost-effectiveness study was done. ACF is a more resource-intensive method of case detection, and its cost-effectiveness, even in high prevalent settings, needs to be assessed [29].

In conclusion, community-based ACF through community health workers, with targeted use of Xpert along smear microscopy proved feasible and effective in poor urban settlements of Phnom Penh. It allowed these communities to timely access TB diagnostic services; it identified a substantial proportion of infectious cases and a flexible patient-centred treatment approach ensured a high treatment success rate. Longer term follow-up data are needed to evaluate the impact on TB transmission. Community-wide TB screening could be considered as a complementary strategy to passive case detection in similar TB-endemic settings.
